# Expected drainage pressures on extracorporeal membrane oxygenation based on in-vitro modeling of pressure-flow variables

**DOI:** 10.1051/ject/2026008

**Published:** 2026-09-15

**Authors:** Yeu Sanz Wu, Caitlin J. Cain-Trivette, Samantha Lai, Caleb Varner, Kenmond Fung, Weijia Fan, Jimmy Duong, Bin Cheng, Nicholas Schmoke, Christopher Nemeh, William Middlesworth, Vincent Duron

**Affiliations:** 1 Division of Pediatric Surgery, Columbia University Irving Medical Center/New York Presbyterian Morgan Stanley Children’s Hospital New York NY USA; 2 Columbia University Vagelos College of Physicians and Surgeons New York NY USA; 3 Department of Clinical Perfusion, New York Presbyterian Morgan Stanley Children’s Hospital New York NY USA; 4 Department of Biostatistics, Columbia University Mailman School of Public Health New York NY USA

**Keywords:** Drainage pressure, Outflow pressure, Circuit flow, Pediatric ECMO, ECMO cannula, Pressure drop, Mathematical model, Intravascular volume

## Abstract

*Background*: Pressure readings used in extracorporeal membrane oxygenation (ECMO) guide a variety of clinical interventions. However, several factors affect the accuracy of these measurements. We aimed to determine a model to mathematically predict expected pressure readings for different pediatric ECMO cannulae. *Methods*: We assembled an in-vitro circuit simulating those used clinically. Flow (mL/min) was adjusted, and the corresponding pressure drop (mm Hg) across the cannula was documented for various Medtronic Bio-medicus^®^ and Life Support^®^ cannulae. We utilized both water and blood product mixtures of differing hematocrit (Hct) levels – analogous to differing viscosities. Experimentally derived mathematical models were then applied to clinical data. *Results*: The relationship between flow and pressure drops across different cannulae and at different Hct levels was graphed and analyzed. At the same flow, larger cannula size corresponded to smaller drops in pressure, and increased viscosity corresponded to larger drops in pressure. Using regression models of the experimental data, we estimated five exponential equations that include Hct and flow to predict the expected pressure drop for different pediatric ECMO cannulae. There was high variability in data among patients, with significant differences between clinically observed and expected pressures. *Conclusion*: Our models derived from in-vitro experimentation are the first steps towards defining the pressure-flow relationship and validating drainage pressure as a surrogate measure of intravascular volume for critically ill patients on ECMO. Further studies are still needed to refine these models and validate their clinical applicability.

## Introduction

Extracorporeal membrane oxygenation (ECMO) remains an integral tool for the management of critically ill children. Since its first reported use in children in the 1970s, its capability of providing life-saving cardiac and respiratory support has led to its increased use in the management of a diverse population of more than 20,800 critically ill adult and pediatric patients each year [1, 2]. Although widely used, the mechanics underlying this technology are intricate and complex, and remain incompletely understood. This knowledge gap contributes to variable interpretation of circuit information into clinical management of these critically ill patients, particularly regarding fluid management strategies [3–5]. With abnormal circulatory physiology and consequent aberrant hemodynamics, fluid management in these patients is challenging. There remains a deficiency of evidence-based clinical parameters to guide fluid therapy for these patients, especially in children [3].

There are parallels between fluid management in ECMO patients and other critically ill patients. Clinicians rely on a variety of clinical parameters representative of a patient’s hemodynamic status to guide fluid therapy [6]. In ECMO patients, an additional parameter that may be used as a surrogate of intravascular volume is the circuit drainage pressure [7]. Drainage pressure is commonly taken to reflect an ECMO patient’s intravascular volume status, especially in the absence of other conventional, but also unreliable, measures like central venous pressure (CVP) [8–10]. An increasingly negative drainage pressure is presumed to be due to hypovolemia, indicating fluid administration [11]. However, hypovolemia is not the only cause of drainage insufficiency [10, 12]. Failure to consider other causes, such as patient agitation, bleeding, vasodilation, tension pneumothorax, cardiac tamponade, intra-abdominal hypertension, or cannula clot or malposition, can contribute to excessive fluid administration [7, 13, 14]. Fluid overload is a common and consequential complication in ECMO patients with significant associated morbidities and mortality [15–18].

Despite its frequent use in clinical assessment of ECMO patients and clinical decisions for volume administration, drainage pressure has yet to be validated as an indicator of intravascular volume status [19]. Available reference data from manufacturers only offer diagrams of pressure drop vs. flow from experiments with water and not blood. In our study, our goal was two-fold. We aimed to better characterize pressure drop across commonly used ECMO cannulae, utilizing blood as the in-vitro circuit fluid, and to model the relationship between pressure, flow, and viscosity. The subsequently derived formula will then allow calculation of the expected pressure drop when variables such as flow and hematocrit are known. If the clinically observed pressure drop is similar to the calculated expected pressure drop, then we propose that the observed drainage pressure in that patient is a reliable marker of intravascular volume. It is not affected by confounding factors like cannula malposition and can be reliably used to manage fluid status in that patient.

## Materials and methods

### In-vitro circuit

**Figure 1:**
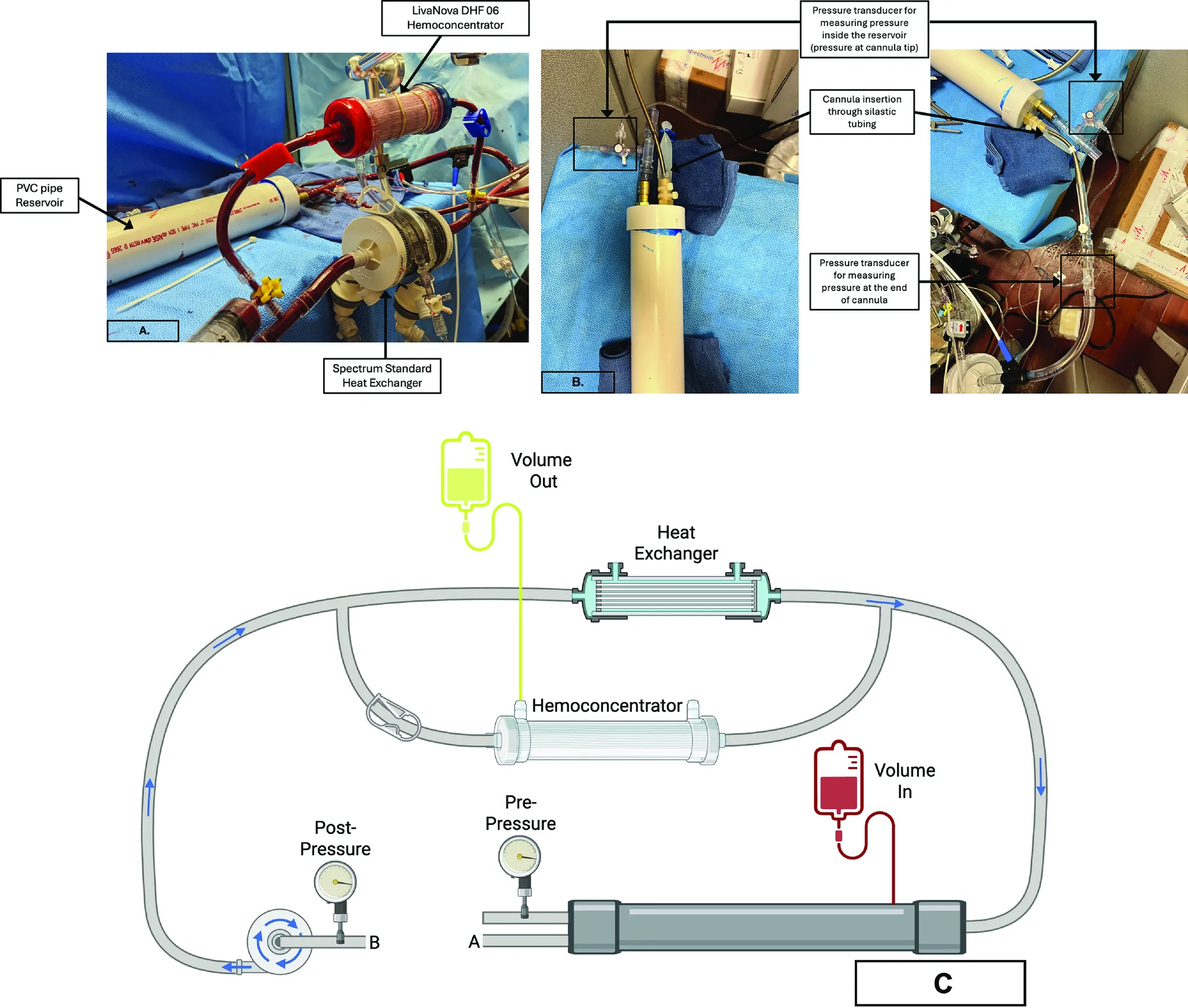
(A) Circuit configuration with hemoconcentrator and heat exchanger. (B) Polyvinyl chloride (PVC) tubing was used for the reservoir with modified ends for cannula insertion and pressure readings.

An in-vitro circuit representative of those used clinically was assembled by a certified clinical perfusionist (CCP) using the Spectrum Quantum CP 37 Centrifugal Drive System (No. CP37V-V0, Spectrum Medical, Mirandola, Italy), the Quantum 12” Workstation Elite^®^ monitoring system (Figure 1A), and 3/8-inch circuit tubing (Figure 1B). The full circuit model is represented in Figure 1C. A LivaNova DHF 06 hemoconcentrator (No. 020119801, LivaNova, Arvada, CO, USA) and Spectrum Standard Heat Exchanger (No. HX55W-S0, Spectrum Medical, Mirandola, Italy) were built into the circuit to allow for manipulation of hematocrit and maintenance of circuit fluid temperature (Figure 1A). A reservoir in which the cannula was inserted and situated consisted of a two-foot-long by two-inch-diameter cylindrical polyvinyl chloride (PVC) pipe with sufficient length to mitigate the effects of recirculation on pressure readings and positioned so that the centrifugal pump and cannulation sites were level. The reservoir dimensions were selected to be long enough to mitigate turbulence in the tube; the smaller length of tubing was chosen in order to decrease usage of donated blood products, given the limited supply. The capped ends were sealed and modified to accommodate three connections; the drainage end consisted of two parallel connections – one allowing for a pressure line to measure the pressure within the reservoir, which presumably corresponded to the pressure at the tip of the cannula, and another to allow for cannula insertion through the side of a silastic tube (Figure 1B). The other end consisted of one connection serving as an inflow to the reservoir. A separate pressure line was attached to the end of the various cannulae to transduce pressure at the end of the cannula. Flow (mL/min) was adjusted on the circuit, and the corresponding pressure drop (mm Hg) was observed and documented following equilibration of the readings. This was done for various flow rates across various cannulae (Figure 1C). The cutoff value for experimentation was based on clinically relevant pressure drop values, which were set at approximately 150 mm Hg.

### Fluids and cannulae

The experiment included Medtronic Bio-medicus^®^ NextGen cannulae (Medtronic, Tijuana, Baja California, Mexico), which are commonly used for pediatric ECMO at our institution. These cannulae included the following sizes: 8 French (Fr), 10 Fr, 12 Fr, 14 Fr, and 19 Fr. We had three different conditions for viscosity: water, hematocrit (Hct) level of 20%, and Hct level of 32%. The first portion of the experiment utilized a water mixture at room temperature (20 °C). This water mixture, which was equivalent to a viscosity of approximately 1 centipoise (cP), or 1 millipascal-second (mPa·s), consisted of a crystalloid mixture of 0.9% normal saline and lactated Ringer’s solution. Total circuit volume was approximately 1.3 L of fluid to account for the reservoir and circuit tubing. Whole blood and platelets were not available for use in the experiment. However, an analogous mixture comprised of expired blood products from the blood bank was utilized following approval from the institutional review board (IRB) (IRB-AAAU9453). To achieve desired hematocrit (Hct) levels of 20% and 32%, 3.5 units of red blood cells (RBCs), three units of fresh frozen plasma (FFP), and 250 mL of 5% (12.5 g) of albumin were mixed into the primed circuit and Hct was measured with epoc^®^ blood analysis system (No. 11413498, Siemens Healthineers, Ottawa, Ontario, Canada) The heat exchanger maintained the blood product mixture in the circuit at normal body temperature (37 °C). The hemoconcentrator allowed for manipulation of the Hct level from 20% to 32%, corresponding to an increase in [20]. Hct levels ranging between 20% to 32% were thought to reflect those seen clinically in [20, 21]. Using the Spectrum 12″ workstation elite, set to flow mode, flow was increased in 50–100 mL increments. Pre- and post-pressure were recorded after allowing flow and pressures to reach steady state. To reduce variability from the mixture, all runs throughout the experiment were completed sequentially on the same day. This allowed for safe storage of the circuit, which was appropriately disposed of following the experiment. Pressure drop was measured using two digital pressure transducers that were connected to the Spectrum 12″ workstation elite. Pressure lines were connected via Luer connections to the circuit, just distal to the cannula (pressure post) and in the reservoir at the tip of the cannula (pressure pre). The resolution of the digital pressure sensors is 1 mmHg. Associated flow and pressure drop were recorded for each cannula size at each hematocrit level.

### Statistical analysis

**Figure 2:**
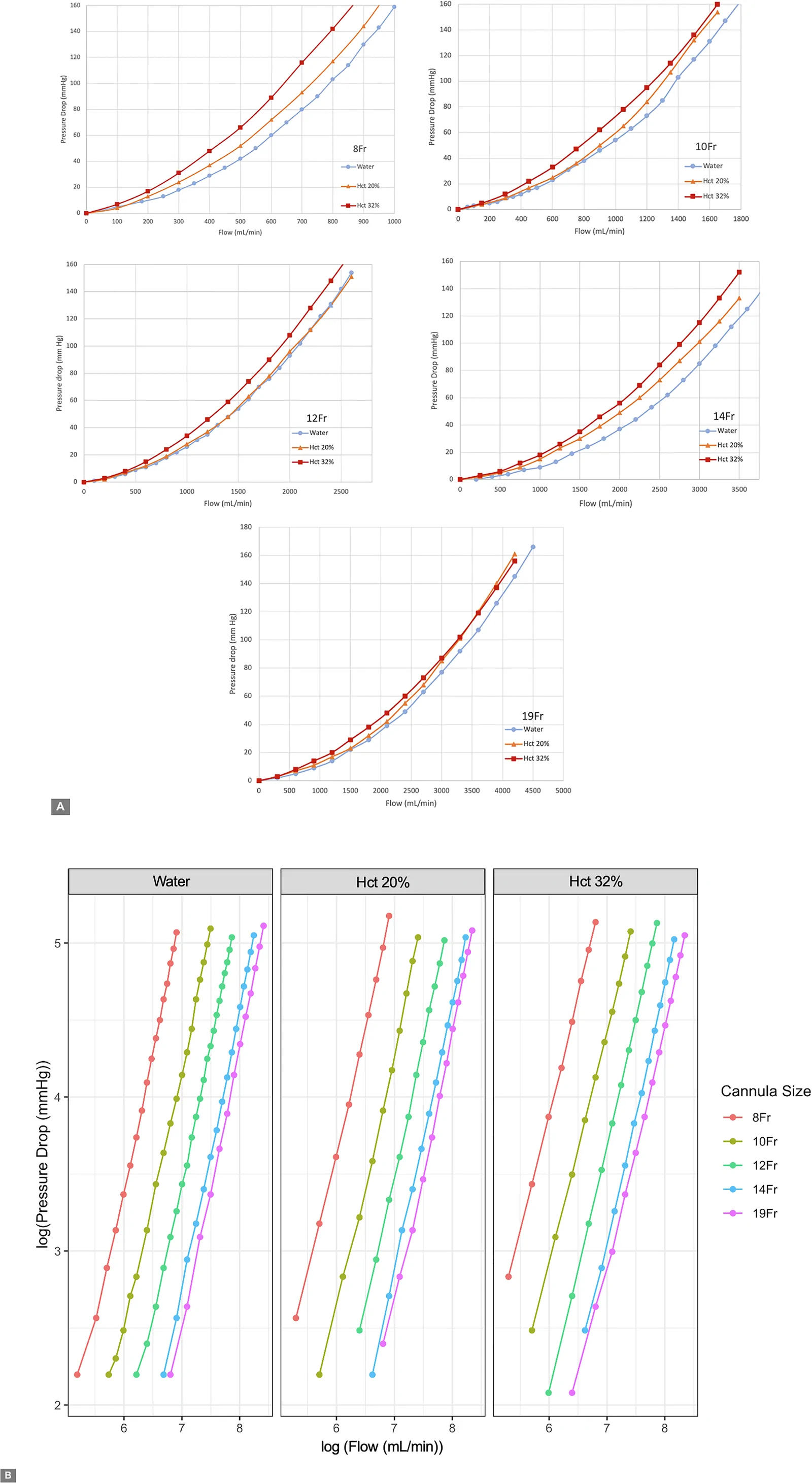
(A) Pressure-flow data plotted and graphed with corresponding trendline for five commonly used Bio-Medicus^™^ venous cannulae of the following sizes: 8, 10, 12, 14, and 19 French (1 Fr equals 1/3 mm). Flow in mL/min on *x*-axis and Pressure drop in mm Hg on y-axis. (B) Log-transformed flow (mL/min) and pressure drop (mmHg) data for different cannulae and at different viscosities – water, hematocrit (Hct) 20%, and Hct 32%. Linear regression models were fitted to this data.

The relationship between flow and pressure drop was stratified by cannula size. Both the flow and pressure drop measurements were transformed using natural log to allow better fitting of linear regression models. Records with natural log-transformed pressure drop of less than two were excluded from the analysis due to less clinically relevant flow, and the resolution of pressure-flow measurements in this range was less accurate. Linear regression models were fitted to quantify the relationship between log-transformed pressure drop and log-transformed flow for each cannula size, as shown in Figure 2B and Table 1. The models also included viscosity (of which there were two values 20% Hct, and 32% Hct) and the interactions between the viscosity and flow. Viscosity is modeled as a continuous variable between 20% and 32%.

**Table 1:** Equations derived from experimental data to describe the expected pressure drop as a function of flow and hematocrit (Hct) for the Hct range of 20–32%.

Cannula size	Model to predict expected pressure drop
8 Fr	p = exp [−7.2729 + 1.7554 ln (flow*) + 0.0608 Hct – 0.0068 ln (flow) × Hct]
10 Fr	p = exp [−9.5929 + 1.9519 ln (flow) + 0.1074 Hct – 0.0137 ln (flow) × Hct]
12 Fr	p = exp [−10.2793 + 1.9192 ln (flow) + 0.0789 Hct – 0.0088 ln (flow) × Hct]
14 Fr	p = exp [−10.5463 + 1.8693 ln (flow) + 0.0607 Hct – 0.0064 ln (flow) × Hct]
19 Fr	p = exp [−12.9971 + 2.1701 ln (flow) + 0.1603 Hct – 0.0197 ln (flow) × Hct]

Fr = French size, approximately 1/3 millimeter; p = pressure drop across cannula (in mm Hg); *Flow in mL/min; Hct = hematocrit (%).

### Clinical application of mathematical models

The experimentally derived equations in Table 1 were applied to clinical data for 47 ECMO patients following separate IRB approval (IRB-AAAR0525). Patient data from the ECMO circuit included CVP, venous flow rate, Hct, and venous pressure. Four patients were excluded due to missing data points. In a clinical setting, drainage pressure (P3) is measured by a transducer connected to the venous/drainage line at approximately 160 cm of tubing from the end of the connection to the cannula. To allow for clinical comparison, pressure drop across this 160-cm length of one-quarter-inch diameter tubing was approximated using Poiseuille’s equation and added to the calculated expected derived pressure drop across the cannula based on cannula size, venous flow rate, and Hct. This total pressure drop was then compared to clinically observed drainage drops, which were determined to be equivalent to clinically observed venous drainage pressures minus CVP. Using the Python library statsmodels, generalized estimating equations with an exchangeable correlation structure were applied to account for multiple observations per patient [22]. Intracluster correlation coefficients (ICC) were calculated to quantify the variation within and between clusters of patient data. A simple linear regression model and an intercept-only model were applied to characterize the relationship between the expected and observed values. Statistical significance was defined as *p* < 0.05.

## Results

### Pressure-flow curves utilizing different viscosity fluids

The experimental data collected of corresponding pressure drops across cannulae at different flow rates were graphed to illustrate the pressure-flow curves for each cannula size. These curves are shown in Figure 2A. When the flow rate (mL/min) was low, the corresponding pressure drop (mm Hg) across the cannula was low. Increasing the flow rate increased the pressure drop exponentially, with a steeper increase noted at higher flow rates. As expected, larger diameter cannulae can accommodate greater flow for the same pressure drop. Thus, pressure drops were higher at lower flow rates in smaller cannulae than in larger cannulae.

When a blood product mixture was utilized in the circuit to assess the effect of viscosity on the pressure-flow relationship, increasing viscosity or increased Hct corresponded to a higher pressure drop at a given flow rate. The effect of viscosity was inconsistent across cannula sizes, warranting the necessity of building a model stratified by cannula size.

### Modeling of the experimental data

Log-transformation allowed for the fitting of linear regression models. Linear regression models were fitted, where the outcome was log-transformed pressure drop and the predictors were log-transformed flow, viscosity, and the interaction between log(flow) and viscosity. This was then back-transformed to provide an equation for predicted pressure drop for each cannula size (Table 1). The expected pressure drops for a specific flow and Hct for each specified cannula size can be calculated based on these models. Given that the model was derived from data taking on values of Hct of 20% or 32%, the interpretation of the model is better restricted to values within this range, and caution should be taken for predictions within this interval too. All models achieved an R-squared of at least 0.99.

### Observed vs. expected pressure differences

**Figure 3:**
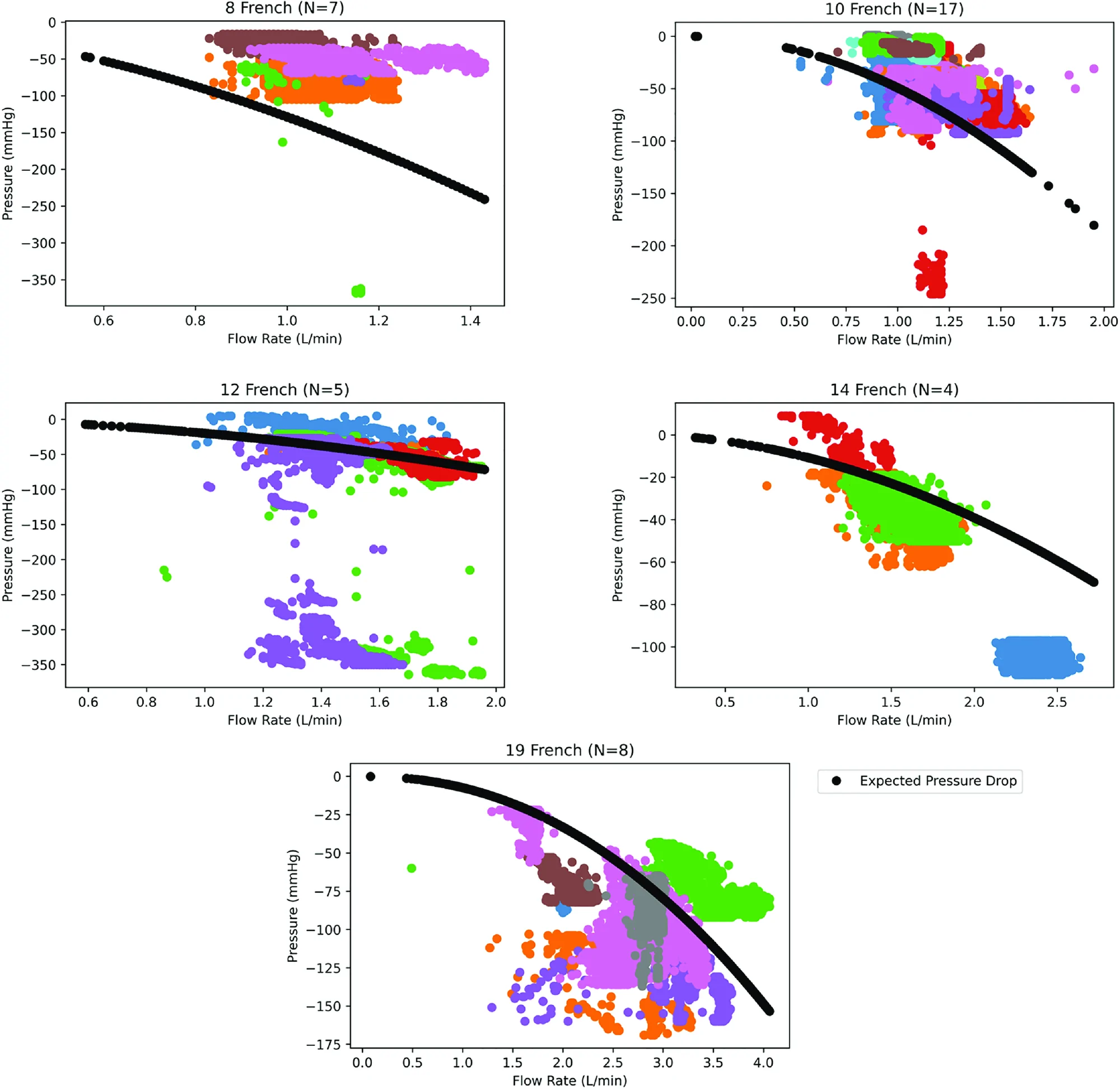
Flow rate (L/min) and pressure (mm Hg) graphs for five commonly used Bio-Medicus^™^ venous cannulae of the following sizes: 8, 10, 12, 14, and 19 French (1 Fr equals to 1/3 mm). Mathematical equations were applied to clinical data to determine the expected drainage pressure (black line) for each flow and hematocrit (Hct). Clinically observed drainage pressure for each patient was plotted, and each color correlated to data from one patient.

The observed and expected pressure drops are plotted in Figure 3. There was a mean number of 5209 datapoints included per patient. ICC between observed and expected drainage pressure among all 47 patients was 0.95 (*p* = 0.002). When assessing ICC between patients with the same cannula size, there remained a significant positive ICC ranging from 0.3 to 0.51. Overall, the mean difference between observed and expected drainage pressures for all patients was −60.6 mmHg (95% CI −72.3, −49.3), *p* < 0.001. Similarly, when analyzed by cannula size, the mean difference between observed and expected remained significantly different, ranging from −92 to −44 mm Hg.

## Discussion

Our experimentally obtained pressure-flow data for various cannulae sizes (in Figure 2A) follow similar curves as those available from the manufacturer, re-demonstrating the recognized inverse relationship between pressure drop and cannula size at the same flow [23]. Our data is in line with prior literature that assesses the pressure–blood flow relation, and like prior experiments, our data may be more clinically applicable and accurate given the usage of a blood product mixture to approximate the viscosity of whole blood [19, 24, 25]. Unlike water, blood is not a simple fluid and is non-Newtonian [26, 27]. Thus, it was important to consider the effect of viscosity on the pressure–flow relationship in our experiment. While a number of previous studies have reported pressure–flow relationships for ECMO cannulae, most data derive either from in vitro systems using water as the test fluid or from manufacturer-provided or purely theoretical models. Our study is unique in that it combines experimentally determined pressure drops from in-vitro circuits using both water and blood-analog solutions with varying hematocrit levels, which more closely simulate patient conditions in pediatric ECMO. Furthermore, whereas prior studies have not systematically integrated clinical data – particularly the subtraction of Central venous pressure (CVP) from observed drainage pressures – we provide direct application of these models to a pediatric clinical population, linking bench results to patient care. This combined approach offers a more physiologically relevant and potentially clinically actionable understanding of drainage pressure variables and supports the validation of drainage pressure as a surrogate marker for intravascular volume across a spectrum of real-world ECMO scenarios [19, 28].

These variables that were utilized in this experiment are from Poiseuille’s equation:

$$∆\text{Pressure}=\frac{8\times \text{viscosity}\times \text{length}\times \text{flow}}{\pi \times r{\text{adius}}^{4}}.$$

This equation is commonly used to describe fluid flow and pressure changes in the field of fluid dynamics. We manipulated the viscosity and radius variables to estimate their effects on pressure change. The experimental data obtained were then used to mathematically characterize the pressure-flow relationship, while also considering viscosity and cannula size, resulting in the exponential equations outlined in Table 1. It is important to highlight that the equations presented are exponential equations. Realistically, blood flow through an ECMO circuit does not strictly follow Poiseuille’s law. This is supported by the fact that when Poiseuille’s equation is utilized to predict pressure drop at a specific flow, the resulting pressure–flow curve is linear, demonstrating that the equation is not a good model for the pressure–flow relationship seen experimentally, or even clinically in in-vivo ECMO circuits. This discrepancy is due to many factors in vitro and even more so in vivo, though not all of which are currently known [24, 29–31].

Poiseuille’s law tends to be accurate in open reservoir systems like cardiopulmonary bypass; however, we acknowledge several limitations in applying Poiseuille’s law to pressure–flow relationships in ECMO circuits. Poiseuille’s law assumes a long, straight, rigid cylindrical pipe with laminar flow of a Newtonian fluid, conditions often violated in clinical ECMO settings. ECMO circuits are closed loops with complex cannula tip geometry, variable vessel anatomy, and blood’s inherent non-Newtonian shear-thinning behavior. These factors contribute to deviations from the idealized linear pressure-flow predictions of Poiseuille’s law observed in our data. Future studies will aim to integrate more advanced fluid dynamic models, such as computational fluid dynamics simulations and mechanistic equations accommodating non-Newtonian flow, to enhance the accuracy and clinical utility of pressure–flow predictions. These efforts will expand on current empirical models to better capture patient-specific and circuit-specific variability, ultimately improving guidance for ECMO management. A more comprehensive understanding of the pressure-flow dynamics of blood flow through the ECMO circuit will allow for a more accurate and reliable clinical application of the large volume of data associated with this technology. We have observed in our current experiment that Poiseuille’s law is not an adequate model to describe flow through an ECMO circuit. Our newly proposed preliminary equations, derived from our experimental data to allow for the prediction of expected pressure drop across different commonly used pediatric ECMO cannulae, require further refinement. When applied to clinical data, our models fall within the range of observed data (Figure 3); however, the high ICC and significant mean differences indicate that there is variation in the data between patients that we have not adequately accounted for. At this stage, we have only explored the effects of known and physically manipulable variables such as radius, viscosity, and flow in an in-vitro study. Additional variables, some that are patient-related, such as vessel geometry and cardiac function, and others, such as suction condition, should also be considered in these equations for an even more accurate prediction of pressure drop [10, 32]. We plan to continue to explore these other variables, particularly in vivo factors, that may affect cannula flow.

An additional limitation of this study is that Reynolds number analysis was not performed. Calculation of Reynolds numbers could provide further insight into the flow regime within the cannulae and help explain deviations from idealized Poiseuille behavior, particularly if transitional or turbulent flow occurred at higher flow rates. Because detailed velocity measurements and specific geometric parameters required for accurate calculation were not prospectively recorded in a manner allowing reliable retrospective derivation, Reynolds numbers could not be calculated for this dataset. Future studies should incorporate prospective Reynolds number analysis to better characterize laminar versus transitional flow conditions during ECMO drainage.

Additionally, while the experimental reservoir was designed to minimize organized flow patterns that could influence pressure measurements, some degree of structured flow within the reservoir may still have occurred. Alternative reservoir configurations, such as larger-diameter junctions or “T” fittings, may further reduce uniform flow characteristics and should be considered in future experimental designs.

Currently, the clinical applicability of these mathematical models remains limited. The scope of our study was a proof-of-concept evaluation to demonstrate that there may be a range of expected and validated drainage pressures for given clinical scenarios (i.e., cannula size, flow rate, Hct). This circuit drainage pressure, also called P3, is already employed by clinicians as a clinical parameter for assessing intravascular volume. However, not only is it affected by several variables, making it imperfect and imprecise, but it is also not yet validated and highly variable.

Our data provide initial guidance for ECMO catheter selection, demonstrating that larger diameter cannulae reliably achieve target flow rates with lower associated drainage pressure, while smaller cannulae incur a higher drainage pressure penalty for comparable flows. By mapping empirically-derived pressure–flow relationships for multiple cannula sizes under pediatric-relevant viscosity conditions, clinicians can select the optimal cannula for a given patient and anticipated flow requirement while minimizing the risk of excessive negative drainage pressures and related complications. This approach helps individualize cannula choices and circuit configurations to patient-specific physiologic and circuit needs and supports more rational fluid management during ECMO.

Our study will eventually enable more accurate and reliable use of drainage pressure and provide reference values to guide clinical assessment of a patient’s intravascular volume on ECMO. This is the first step towards validating drainage pressure as a surrogate measure of intravascular volume and ultimately improving fluid management strategies for these patients.

## Data Availability

Data available on request from the authors.
